# Docetaxel loaded mPEG-PLA nanoparticles for sarcoma therapy: preparation, characterization, pharmacokinetics, and anti-tumor efficacy

**DOI:** 10.1080/10717544.2021.1945167

**Published:** 2021-06-28

**Authors:** Jianhua Chen, Eerdunbagena Ning, Zhijun Wang, Ziqi Jing, Guijie Wei, Xue Wang, Pengkai Ma

**Affiliations:** a School of Chinese Materia Medica, Beijing University of Chinese Medicine, Beijing, China; b Haikou People’s Hospital, Hainan, China; c Division of interventional radiology, Department of Geriatric Medicine &National Clinical Research Center of Geriatric Disease, The 2nd Medical Center of Chinese PLA General Hospital, Beijing, China; d Department of interventional radiology, The 1st & 5th Medical Center of Chinese PLA General Hospital, Beijing, China

**Keywords:** Docetaxel, polymer nanoparticle, sarcoma, pharmacokinetics, pharmacodynamics

## Abstract

Sarcoma represents one of the most common malignant tumors with poor treatment outcomes and prognosis. Docetaxel (DTX) is acknowledged as one of the most important chemotherapy agents. The aim of this study was to improve the efficacy of docetaxel by incorporation into the mPEG-PLA nanoparticle (DTX NP) for the treatment of sarcoma. The DTX NP was prepared by emulsion solvent diffusion method and the prescription and preparation process were optimized through a single factor experiment. The optimized DTX NP was characterized by drug loading, encapsulation efficiency, drug release, etc. Then, the pharmacokinetics was conducted on rats and tumor-bearing ICR mice. Finally, the anti-tumor efficacy of DTX NP with different dosages was evaluated on tumor-bearing ICR mice. The optimized DTX NP was characterized by around 100 nm sphere nanoparticles, sustained *in vitro* drug release with no obvious burst drug release. Compared with DTX injection, the AUC of DTX NP increased by 94.7- and 35.1-fold on the rats and tumor-bearing ICR mice models, respectively. Moreover, the intra-tumoral drug concentration increased by 5.40-fold. The tumor inhibition rate of DTX NP reached 94.66%, which was 1.24 times that of DTX injection (76.11%) at the same dosage, and the bodyweight increase rate was also higher than the DTX injection. The study provided a DTX NP, which could significantly improve the bioavailability and therapeutic efficacy of DTX as well as reduced its toxicity. It possessed a certain prospect of application for sarcoma treatment.

## 1. Introduction

As one of the most frequent primary malignancies, sarcoma has been a profound health threat to children and adolescents (Ray-Coquard et al., 2018). Surgery is still the first choice for sarcoma therapy, and its 5-year overall survival rate has increased to near 80%. However, due to the minimal residual tumor cells within the surgical field, tumor recurrence and metastasis (especially bone and lung) has been the major factor that limits the extension of patient survival. Thus, chemotherapy is an important approach to increase the preoperative limb salvage rate and decrease the postoperative tumor recurrence rate (Gamboa et al., 2020).

Docetaxel (DTX) is a semi-synthetic product of a renewable resource, the needles of the European yew (*Taxus baccata* L.), belongs to the second generation taxoids antitumor drug (Ojima et al., 2018). Though structurally similar to paclitaxel, DTX not only has a wider spectrum of anticancer activity but also is more potent as an inhibitor of microtubule depolymerization (Riccardi et al., 1995). At present, DTX injection (Taxotere^®^) is the sole commercial DTX dosage form that is used for treating advanced breast cancer, non-small cell lung cancer, gastric cancer, ovarian cancer, etc (Petronille et al., 2020). However, the severe adverse effects of DTX injection have greatly limited its further application. On one side, the nonspecific distribution of DTX commonly induces myelosuppression, neurotoxicity, cardiovascular toxicity, and gastrointestinal reaction; on the other side, an amount of Tween-80 and ethanol are served as co-solvent due to the high lipophilicity of DTX, which causes a series of allergic reactions (Chanat et al., 2015; Ben Nasr et al., 2021). Therefore, there is an urgent need for developing a new formulation to improve the solubility and tumor targeting of DTX.

Nowadays, nano-drug delivery system has been an important approach to improve drug solubility, tumor-targeting ability, and reduce drug toxicity. The lipophilic drug can be encapsulated in the nano-sized particle to improve its water solubility. And due to the abnormalities of tumor vasculature and lymphatic vessels, the nanoparticle can accumulate in the tumor through the well-known ‘EPR effect’ (Fang et al., 2020). Further coating or conjugating with hydrophilic polymers, such as polyethylene glycol (PEG), hyaluronic acid, and chitosan, the nanoparticles can avoid absorption with protein thus prolonging its circulation *in vivo* and enhancing accumulation in tumor (Tehrani et al., 2019). Based on this, a variety of DTX nano-drug delivery systems has been designed, such as liposome (Chen et al., 2017), micelle (Ghamkhari et al., 2019), cyclodextrin inclusion complex (Ren et al., 2020), etc. Among these, the polymer-based nanoparticle has a unique advantage for its easy preparation, high stability, and flexible regulation. Particularly, several biocompatible polymers (PLGA, PLA, PCL) have been approved by FDA for tissue engineering, medical material, and drug carrier (Tipnis & Burgess, 2018). The degradation products of these polymers can be metabolized into carbon dioxide and water through the tricarboxylic acid cycle and excreted by the kidneys (Park et al., 2019). Inspiringly, Genexol-PM (a paclitaxel-loaded PEG-PLA micelle) has been approved in South Korea for the treatment of metastatic breast cancer non-small cell lung cancer, and ovarian cancer (Kim et al., 2017). The replacement of Cremophor EL significantly reduces severe side effects of the injection.

This study intended to prepare a DTX loaded PEG-PLA nanoparticle (DTX NP) for sarcoma therapy. Firstly, the DTX could be encapsulated in the nanoparticle to improve its solubility and avoid using the Cremophor EL as a solvent; then, the PEG shell of DTX NP could realize long-circulating and targeted to tumor through the ‘EPR effect’; finally, the DTX was sustained release from DTX NP with degradation of PLA to maintain effective therapeutic concentration long time. Thus, the DTX NP holds promising in enhancing the therapeutic outcome and reducing the adverse effects of DTX.

## 2. Materials and method

### 2.1. Materials and animals

Docetaxel was purchased from Alfa Aesar (Shanghai, China). Poly(ethylene glycol)-b-poly(lactic acid) (mPEG-PLA, MW = 13,000 ∼ 100,000) and polylactic acid (PLA, MW = 5000) were purchased from Daigang Biomaterial Co., Ltd (Jinan, Shandong Province, China). Commercial docetaxel injection (Duopafei^®^) was acquired from Qilu Pharmaceutical Co., Ltd (Jinan, Shandong Province, China). Tween-80 and (2-Hydroxypropyl)-*β*-cyclodextrin were purchased from Sinopharm Chemical Reagent Co., Ltd (Beijing, China). Sodium cholate was purchased from Sigma-Aldrich (St. Louis, MO, USA). Polyvinyl alcohol was purchased from Kuraray Co., Ltd (Osaka, Osaka Prefecture, Japan). Cyclophosphamide for injection was purchased from Hengrui Pharmaceuticals Co., Ltd (Lianyungang, Jiangsu Province, China). All chemicals obtained were used without additional purification. Tumor-bearing nude mice (20 ± 2 g) and rats (200 ± 20 g) were purchased from Zhonghongboyuan Biotechnology Co., Ltd (Beijing, China). All animal experiments were conducted with the Guide for the Care and Use of Laboratory Animals of Beijing University of Chinese Medicine.

### 2.2. Preparation of DTX-NPs

Docetaxel-loaded mPEG-PLA nanoparticles were prepared by the emulsion solvent diffusion method (Jia et al., 2008). Briefly, docetaxel (DTX) and polyethylene glycol-polylactic acid (mPEG-PLA) was dissolved in the mixed solvent (ethyl acetate/benzyl alcohol, 1/1) to form the organic phase. An appropriate amount of emulsifier was dissolved in the mixed solvent (water/ethyl acetate/benzyl alcohol, 10/1/1) to form the aqueous phase. The organic phase was dropwise added into the aqueous phase and homogenated for half an hour to form a coarse emulsion. The coarse emulsion was granulated by a homogenizer (Emulsiflex-C3, Avestin, Canada) to form the mini emulsion with uniform particle size. The mini emulsion was dropped into water at 4 °C and solidified to form a nanoparticle suspension. Finally, the unencapsulated drug was removed by ultrafiltration to obtain the docetaxel mPEG-PLA nanoparticles. Taking particle size and encapsulation rate as indexes, the concentration of organic phase (5%, 10%, 20%, and 50%), type and concentration of emulsifier (0.1%, 0.5%, 1% sodium cholate, and 1% polyvinyl alcohol), volume ratio of the organic phase and water phase (1:2, 1:5 and 1:10), the strength of homogeneity (5000, 7500, 10,000, 12,500 and 15,000 psi), molecular weight of mPEG-PLA polymer (M 13,000–100,000), the addition of small molecular weight PLA (M 5000), lyophilization conditions and lyophilization protectants (glucose, sucrose, trehalose, and *β*-cyclodextrin) were investigated to obtain the optimal prescription and preparation process.

### 2.3. Characteristic of DTX-NPs

DTX-NPs was diluted with purified water to an appropriate concentration (10 mg/mL). The zeta potential, particle size, and distribution of DTX-NPs were measured using a particle size analyzer (NANO-ZS, Malvern, England). The morphology of DTX-NPs were observed by the TEM (JEM-2100F, JEOL, Japan).

Ultrafiltration method was used to determine the encapsulation efficiency and drug loading (Chen et al., 2016). Briefly, appropriate amount of Tween-80 was added in the nanoparticle suspension, stirred to dissolve free DTX, and then transferred in an ultrafiltration membrane bag (300 kDa, Labscale, Millipore Corporation, USA) and dialyzed water to separate free drugs. After ultrafiltration, the DTX-NPs were collected and dissolved with methanol. The content of the drug was determined with high-performance liquid chromatography (1200, Agilent, USA). Chromatographic determination conditions: column, Kromasil C18 (4.6 × 150 mm, 5 μm); mobile phase, acetonitrile:water = 50:50 (v/v); flow rate, 1.0 mL/min; column temperature, 30 °C; injection volume, 20 µL; detection wavelength, 230 nm. The encapsulation efficiency (EE%) and drug loading (DL%) were calculated with the following formula: EE% = *W_n_*/*W_t_* × 100%; DL% = *W_n_*/(*W_n_*+*W_p_*) × 100%. (*W_n_*, drug encapsulated in nanoparticles; *W_t_*, the total drug in nanoparticle suspensions; *W_p_*, the amount of polymer in nanoparticles).

The membrane dialysis method was used to investigate the *in vitro* drug release. 2 mL DTX-NPs prepared from different molecular polymers were packaged in a dialysis bag, immersed in 150 mL release medium (0.02 M pH 7.4 PBS buffer with 1% *β*-cyclodextrin), and stirred at 300 rpm. At a predefined time point, 1 mL release medium was pipetted out for HPLC determination to calculate the cumulative release percentage, and another 1 mL blank release medium was supplied in the release medium at the same time.

### 2.4. Pharmacokinetics in rats

Eighteen rats were randomly divided into three groups (*n* = 6), and were injected by tail vein with commercially available DTX-injection, DTX-NPs1 (mPEG_5000_PLA_15000_) and DTX-NPs2 (mPEG_5000_PLA_15000_ with 40% PLA_5000_) at a single dose of 5 mg/kg. The rats were anesthetized with ether and the blood samples (0.5 mL) were collected at 0.083, 0.25, 0.5, 1, 2, 4, 8, 24, 48, 72 and 96 h post-administration. The blood samples were placed in heparinized tubes, centrifuged at 8000 rpm for 5 min, and the supernatants were stored at −80 °C. 150 μL serum and 15 μL internal standard solution (paclitaxel) was transferred into a 10 mL centrifuge tube. The mixture was extracted with 3 mL tert-butyl ether and vortexed for 3 min, then centrifuged at 2000 rpm for 10 min. The supernatant (2 mL) was placed in a water bath (40 °C) and evaporated to dryness under a gentle flow of nitrogen. Afterward, the residues were re-dissolved in 200 μL mobile phase, vortexed for 2 min, and centrifuged (12,000 rpm) for 10 min. 10 μL supernatant was injected for LC/MS/MS analysis (API4000, ABSCIEX, USA). The pharmacokinetic parameters were calculated using the DAS3.0.8 software.

The deternimation parameters were as follow. Chromatographic conditions: Chromatographic column, Symmetry C18 (5 µm, 3.9 × 150 mm, Waters); Mobile phase, 0.5% formic acid aqueous solution/0.5% formic acid methanol solution (20:80, V/V); flow rate, 0.5 mL/min; column temperature, room temperature; injection volume, 10 µL; Mass spectrometry conditions, electrospray ion source (ESI); ionization mode, positive ion ionization; scanning method, multiple reaction monitoring mass spectrum; ionspray voltage, 5500.0 V; atomizer temperature, 100 °C; curtain gas (CUR), 10 psi; collision gas (CAD), 8 psi; docetaxel ion pair, 808.5/527.3 (collision energy: 14 eV), 808.5/226.1 (CE: 20 eV); clustering voltage DP, 60 V; internal standard ion pair, 854.4/569.4 (CE: 16 eV), 854.4/286.2 (CE: 26 eV); DP, 95 V.

### 2.5. Pharmacokinetics and tumoral drug distribution in tumor-bearing mice

The tumor-bearing ICR mice model was established as described previously (Yu et al., 2019). The logarithmic phage of S180 tumor cells was digested with trypsin, collected under aseptic operation, and diluted with normal saline into 1:4 cell suspension. 0.2 mL cell suspension was subcutaneously inoculated in the right forearm armpit of ICR mice. They were randomly divided into 3 groups (*n* = 6), which were DTX injection, DTX-NP_S_1, and DTX-NP_S_2, respectively. Following administration through tail vein at a dose of 50 mg/kg, blood samples were collected at 0.083, 0.25, 0.5, 1, 2, 4, 6, 8, 10, 24, 48 and 72 h, respectively. Then, the mice were sacrificed, and the tumor was removed and weighed. A piece of tumor tissues was cut off, added with 5 times the volume of normal saline, and homogenized with a high-speed electric homogenizer. 150 µL tissue suspension was added with 15 μL internal standard solution, extracted with 3 mL tert-butyl ether, centrifuged at 2000 rpm for 10 min, 2 mL of the supernatant was pipetted out and dried with N_2_. The residue was re-dissolved in 200 μL mobile phase, vortexed for 2 min, centrifugated at 12,000 rpm for 10 min. 10 μL supernatant was determined with the LC-MS method.

### 2.6. Anti-tumor efficacy

The tumor-bearing mice were randomly grouping, which was model group, saline group, cyclophosphamide group, DTX injection group, DTX-NPs1 (mPEG_5000_PLA_15000_), DTX-NPs2 (mPEG_5000_PLA_20000_), and DTX-NPs3 (mPEG_5000_PLA_28000_) groups at low, medium, and high doses (4, 8 and 16 mg/kg) (*n* = 10). The mice were administered once every two days for a total of 3 times. Following tumor growth of about 1 g, the mice were sacrificed and the tumor was cut off and weighed. The tumor inhibition rate was calculated by the following formula: tumor inhibition rate = (1 − *T*/*C*) × 100%, (*T*, the average tumor weight of the treatment group; *C*, the average tumor weight of the model group). During the experiment, the animals’ appearance, behavioral activities, fecal traits, and local drug reactions were observed and recorded. The bodyweight of mice was monitored to evaluate the effect on the growth of mice

### 2.7. Statistical analysis

The experimental data were expressed as mean values and standard deviation. The statistical test was carried out by the one-way ANOVA method with PASW 18.0 statistical software. The difference was significant when *p* < .05, and the difference was extremely significant when *p* < .01.

## 3. Results and discussion

### 3.1. Optimization of DTX-NPs

The prescription and preparation process of DTX NPs was detailed investigated. As shown in Table 1, the concentration of organic phase, the type, and concentration of emulsifier, the volume ratio of organic phase and water phase, homogeneous strength, the molecular weight of the polymer, and addition of small molecule polymer were key factors for the preparation of DTX NPs. Analyzing the results of the single-factor method, the optimal prescription and preparation process was as follows: drug/polymer ratio, 250 mg DTX, and 1000 mg polymer; the organic phase, 2 mL ethyl acetate mixed with 0.5 mL benzyl alcohol; aqueous phase, 12.5 mg sodium cholate dissolved in 12.5 mL water, ethyl acetate, and benzyl alcohol mixed solvent; macroemulsion, 10,000 rpm for half an hour; homogenization intensity, 5000 psi; solidification, 30 times volume of cold water at 4 °C.

**Table 1.** Single factor analysis of the DTX-NPs preparation.

Formulation variables		EE (%)	Size (nm)	PDI
Concentration of organic phase	5%	9.35	70.10	0.589
	10%	15.75	83.17	0.503
	20%	29.60	96.83	0.247
	50%	38.14	104.4	0.093
Emulsifier	No	39.40	112.0	0.209
	SDC 0.1%	39.67	99.26	0.088
	SDC 0.5%	35.65	94.47	0.216
	SDC 1%	20.33	73.92	0.239
	PVA 1%			
Organic phase/water phase	1:2	2.06	102.0	0.109
	1:5	39.67	99.26	0.088
	1:10	32.32	96.47	0.114
Homogeneous pressure (psi)	4000		118.4	0.140
	5000		106.2	0.166
	7500		124.4	0.135
	10,000		258.3	0.364
Molecular weight of polymer	5000–8000	13.07	93.00	0.257
	5000–12,000	19.57	90.07	0.167
	5000–15,000	35.65	94.57	0.206
	5000–20,000	35.69	99.01	0.147
	5000–28,000	39.04	99.50	0.127
	5000–95,000		225.2	0.325
mPEG-PLA%	80% +0%	38.14	104.4	0.093
+ PLA (5000)%	40%+40%	61.88	156.5	0.097

The influence of freeze-drying protective agents on the quality of DTX NPs was also investigated (Table 2). When protective agents containing 20% cyclodextrin, the freeze-dried products possessed a favorable appearance, good re-solubility, and uniform particle size. Therefore, it was necessary to add cyclodextrin as freeze-drying protective agents for freeze-dried products.

**Table 2.** The effect of cryoprotectants on DTX-NPs.

Lyophilized protective agent	Appearance	Re-dispersibility	Particle size (nm)	PDI
No	Collapsed	Poor		
20% glucose	Collapsed	Good	123.6	0.238
20% sucrose	Even and full	Poor		
20% trehalose	Even and full	Poor		
20% cyclodextrin	Even and full	Good	100.9	0.083
10% sucrose + 10% cyclodextrin	Delamination, partial collapse	Good	101.9	0.123
10% trehalose + 10% cyclodextrin	Delamination, partial collapse	Good	102.3	0.078

### 3.2. Characteristics of DTX NPs

**Figure 1.**
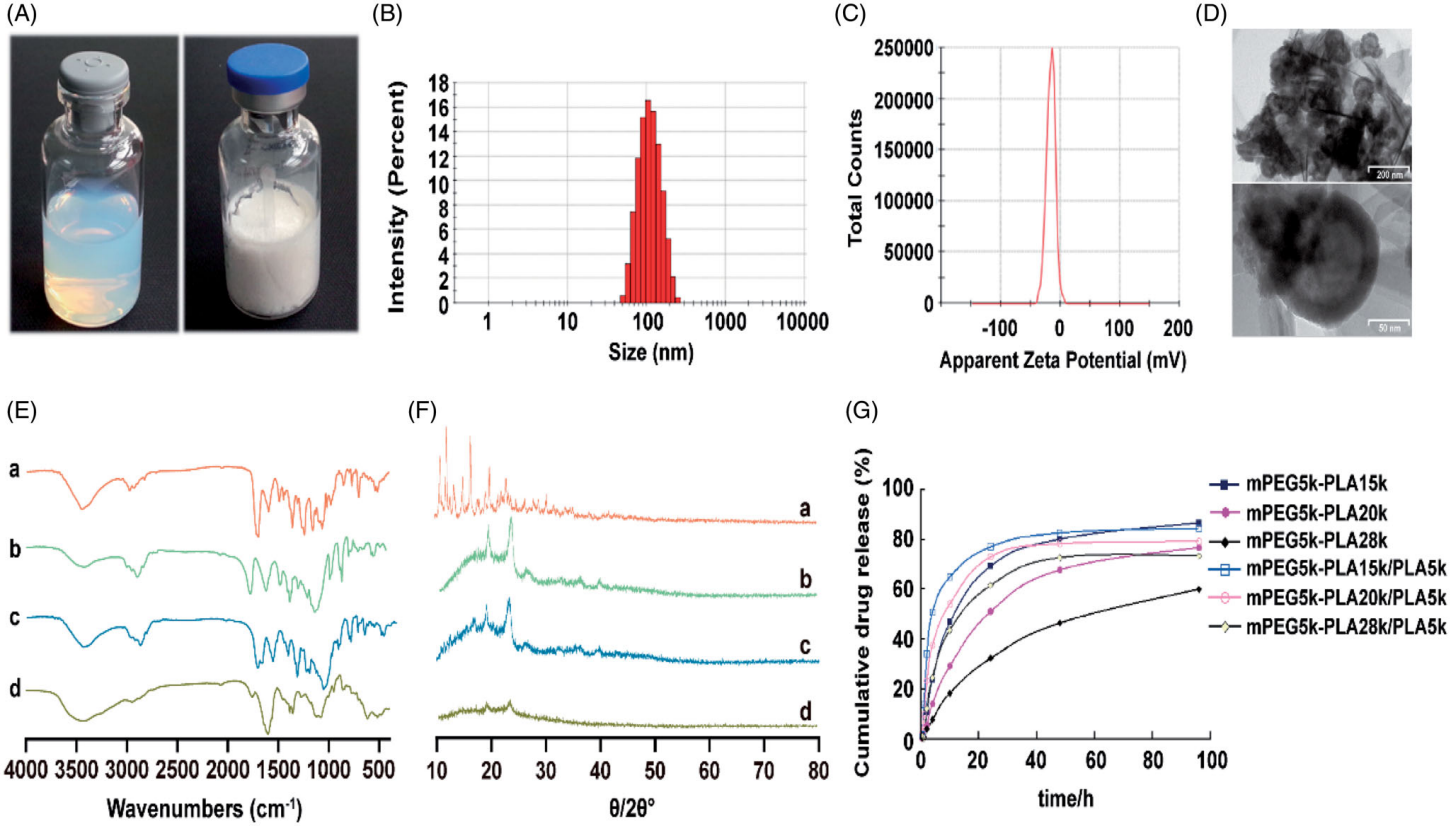
(A) The picture of DTX-NPs suspension and freeze dried sample; (B) The particle size distribution of DTX-NPs; (C) The zeta potential distribution of DTX-NPs; (D) TEM images of DTX-NPs; (E, F) IR and XRD spectra of (a) DTX, (b) mPEG-PLA, (c) physical mixture of DTX and mPEG-PLA, (d) DTX-NPs; (G) The *in vitro* drug release curve of DTX-NPs.

The freeze-dried DTX NP presented loosely round cake-like powder and easily re-dispersed in water, and the DTX NP suspension was translucent emulsion with light blue opalescence (Figure 1(A)). The particle size was 105.7 ± 1.2 nm, and possessed narrow distribution (PDI < 0.2), demonstrating the particle size was uniform (Figure 1(B)). The average zeta potential of the DTX NP was −16.47 ± 0.38 mV, showing weak electronegativity (Figure 1(C)). Besides, the TEM observation confirmed its sphere morphology and approximate 100 nm particle size (Figure 1(D)). Due to the DTX NP was around 100 nm and negative charge, so it could avoid adsorption by plasma proteins and eliminating by the reticuloendothelial system, as well as easily passive targeting the tumor through the ‘EPR effect’ (Bewersdorff et al., 2020).

As IR and XRD spectra showed in Figure 1(E,F), mPEG-PLA shared characteristic absorption peaks: *ν*_OH_(3456.29 cm^−1^), *ν*_CH3_(2994.98 cm^−1^), *ν*_CH2_(2946.40 cm^−1^), *ν*_CH_(2890.70 cm^−1^), *ν*_C=O_(1758.27 cm^−1^), *δ*_CH2_(1456.08 cm^−1^), *δ*_CH3_(1382.06 cm^−1^), *β*_OH_(1363.25 cm^−1^), *ν*_C-O_(1113.46 cm^−1^), which still existed in the physical mixture while disappeared in the DTX NPs. DTX shared characteristic absorption peaks: *ν*_C=C_(1601.23, 1494.54, 1452.25 cm^−1^); *ν*_C=O_(1704.91 cm^−1^), which also disappeared in the DTX NPs.DTX had crystal diffraction peaks in the range of 10°–30°, which disappeared in the physical mixture and DTX NPs. And the peak intensity of DTX NPs decreased compared with the physical mixture. It could be inferred that DTX encapsulated in DTX NPs was in the amorphous phase and formed intermolecular interactions (hydrogen-bond, van der Waals force, etc.) with the mPEG-PLA polymers (Kang et al., 2017).

The *in vitro* drug release curve was shown in Figure 1(G), DTX-NPs prepared from different molecular polymers all could continuously release drug for more than 48 h, and there was only no more than 2.61% drug release in 0.5 h, indicating that the DTX-NPs had obvious sustained drug release effect and no initial burst release. The drug release rate slowed down with the increase of polymers’ molecular weight. Theoretically, the degradation rate of the polymer decreased with the increase of its molecular weight that slowed the drug release rate (Bohr et al., 2017). So, the drug release profile in the study was in accordance with the theory. The addition of PLA_5000_ had a significant influence on the drug release rate, it could significantly accelerate the drug release of DTX-NPs. For the DTX-NP (mPEG_5000_PLA_15000_), the cumulative drug release of the DTX-NP with PLA_5000_ in 4 h was 50.88%, while it was only 23.71% for the DTX-NP without PLA_5000_. It might be caused by the faster degradation rate of low molecular PLA than the high molecular PLA. Therefore, the drug release rate could be controlled by changing the molecular weight of the polymer or adjusting the ratio of PLA_5000_.

### 3.3. Pharmacokinetics of DTX-NPs in normal rats

**Figure 2.**
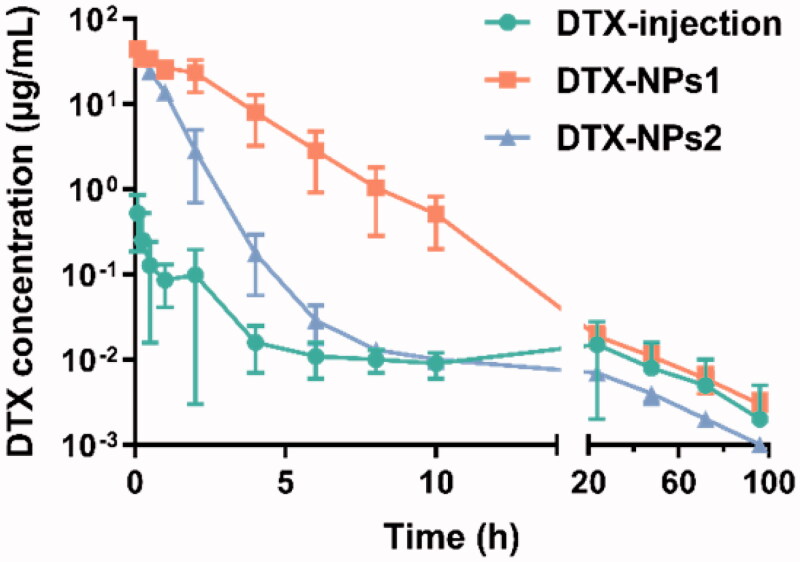
Blood concentration-time profiles of DTX in rats after intravenous administration of DTX-NPs (*n* = 6).

The average blood drug concentration-time curve was shown in Figure 2. It could be seen from the results that after administration of the DTX injection, the drug concentration decreased rapidly, while the DTX NPs could maintain a higher drug concentration than the DTX injection for a certain period of time. The addition of small molecule PDLLA accelerated the elimination of the drug, which might be related to its faster drug release rate. The main pharmacokinetic parameters were summarized in Table 3. The maximum blood concentration *C*_max_ of the nanoparticle preparations was 44.374 and 43.663 mg/L, which was significantly higher than that of the DTX injection (0.525 mg/L). The AUC of the DTX-NPs1 and DTX-NPs2 preparations were 94.7 and 33.1 times that of the DTX injection, respectively, indicating the bioavailability of the DTX was significantly improved due to sustained drug release from DTX NPs.

**Table 3.** The main pharmacokinetic parameters of DTX in rats after intravenous administration of DTX-NPs (*n* = 6).

Parameter	DTX-injection	DTX-NPs1	DTX-NPs2
AUC(0–t) (mg/L h)	1.176 ± 0.57	111.374 ± 29.301	38.89 ± 3.145
AUC(0–∞) (mg/L h)	1.283 ± 0.699	111.492 ± 29.353	38.917 ± 3.141
AUMC(0–t) (h h mg/L)	28.467 ± 26.724	289.599 ± 101.896	40.003 ± 7.76
AUMC(0–∞) (h h mg/L)	43.322 ± 48.063	305.64 ± 109.57	43.423 ± 7.253
MRT(0–t) (h)	21.275 ± 10.422	2.555 ± 0.312	1.024 ± 0.132
MRT(0–∞) (h)	27.949 ± 16.737	2.691 ± 0.353	1.112 ± 0.121
*t*_1/2z_ (h)	24.52 ± 5.214	25.886 ± 2.658	22.065 ± 1.469
*T*_max_ (h)	0.083 ± 0	0.083 ± 0	0.083 ± 0
*V_z_* (L/kg)	173.605 ± 104.2	1.731 ± 0.292	4.129 ± 0.621
CL_z_ (L/h/kg)	5.258 ± 3.693	0.047 ± 0.012	0.129 ± 0.011
*C*_max_ (mg/L)	0.525 ± 0.34	44.374 ± 7.023	43.663 ± 7.96

### 3.4. Pharmacokinetics of DTX-NPs in tumor-bearing mice

**Figure 3.**
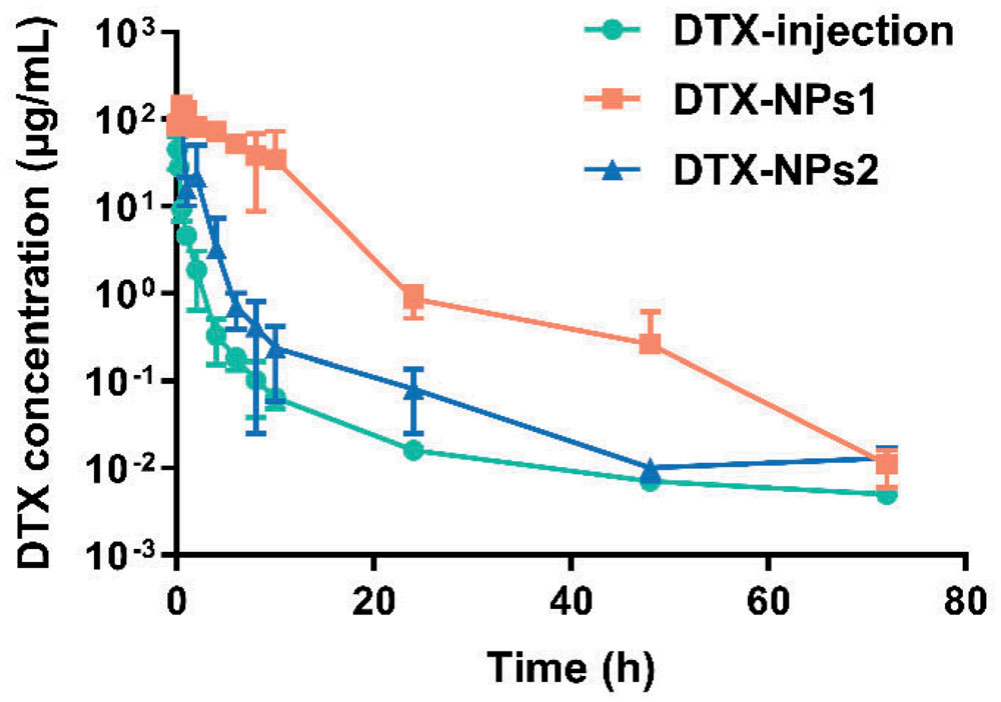
Blood concentration-time profiles of DTX in bearing-mice after intravenous administration of DTX-NPs (*n* = 6).

Because the pharmacokinetic changed greatly among different species (Fontaine et al., 2019), so the pharmacokinetic of DTX-NPs was also evaluated in tumor-bearing mice. As shown in Figure 3, the plasma drug concentration of DTX injection was 44.8 μg/mL at 5 min after administration, and then decreased rapidly to lower than 1 μg/mL 2 h later. For the DTX-NPs1, the plasma concentration was 84.9 μg/mL at 5 min and continuously elevated for about half an hour, and then decreased to below 1 μg/mL after 24 h. For the DTX-NPs2, its plasma drug concentration directly decreased from 115.4 μg/mL to 1 μg/mL during 6 h. The elimination rate sequence in mice was: DTX injection > DTX-NPs2 > DTX-NP1, indicating that nanoparticles significantly slowed down the elimination of drugs. Compared with the DTX-NPs1, the DTX-NPs2 formulation with PDLLA exhibited a faster elimination rate, which was consistent with the *in vitro* drug release profile and pharmacokinetics in rats. The main pharmacokinetic parameters were shown in Table 4. The AUC of DTX-NPs1 and DTX-NPs2 was 35.1 and 5.2 times higher than the commercial preparations, indicating that nanoparticles also could significantly improve the bioavailability of the drug in tumor-bearing mice.

**Table 4.** The main pharmacokinetic parameters of DTX in bearing-mice after intravenous injection (*n* = 6).

Parameters	DTX injection	DTX-NP1	DTX-NP1-1
AUC(0–t) (mg/L h)	25.873 ± 5.044	908.454 ± 241.281	134.663 ± 46.558
AUC(0–∞) (mg/L h)	26.048 ± 5.136	908.575 ± 241.216	134.742 ± 46.66
AUMC(0–t) (h h mg/L)	44.504 ± 8.017	5618.552 ± 2706.717	221.314 ± 131.249
AUMC(0–∞) (h h mg/L)	65.001 ± 25.036	5628.598 ± 2701.818	228.685 ± 141.481
MRT(0–t) (h)	1.738 ± 0.22	5.951 ± 1.406	1.558 ± 0.382
MRT(0–∞) (h)	2.46 ± 0.663	5.963 ± 1.4	1.601 ± 0.424
*t*_1/2z_ (h)	25.534 ± 10.314	7.382 ± 0.96	10.667 ± 3.551
*T*_max_ (h)	0.125 ± 0.083	0.75 ± 0.289	0.188 ± 0.208
*V_z_* (L/kg)	69.135 ± 20.255	0.628 ± 0.212	5.97 ± 1.923
CL_z_ (L/h/kg)	1.988 ± 0.466	0.058 ± 0.014	0.403 ± 0.125
*C*_max_ (mg/L)	46.162 ± 15.693	146.889 ± 51.243	116.999 ± 8.625

**Figure 4.**
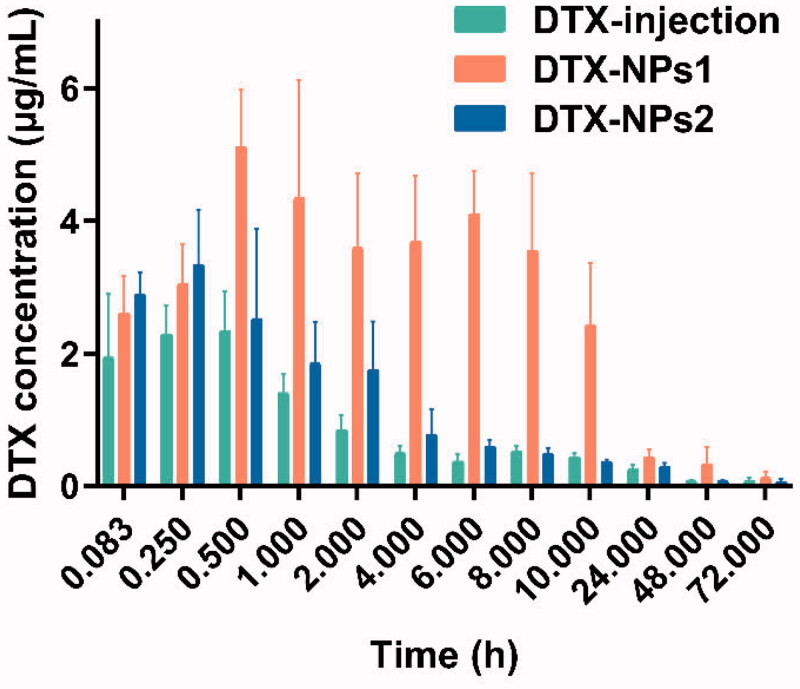
Concentration of DTX in tumor-bearing mice at different time (*n* = 6).

The intra-tumoral drug concentration-time curve was shown in Figure 4, the *C*_max_ of DTX injection group was 1.946 µg/mL at 5 min after administration and rapidly declined to 1 µg/mL 2 h later. For DTX-NPs1, the *C*_max_ was 2.598 µg/mL, and fluctuated within the range of 2.4 ∼ 5.2 µg/mL for 10 h, which could maintain a high drug level for a long time. Besides, the DTX-NPs1 showed significantly higher drug concentration than the DTX-NPs2 after 0.25 h. Thus, there was a similar tendency between the intra-tumoral drug concentration–time curve and blood drug concentration–time curve. The higher concentration and slower elimination rate of the intra-tumoral drug for DTX-NPs, compared with the commercial injection, was beneficial for enhancing the anti-tumor effect and reducing side effects of DTX.

### 3.5. Anti-tumor effect of DTX-NPs

**Figure 5.**
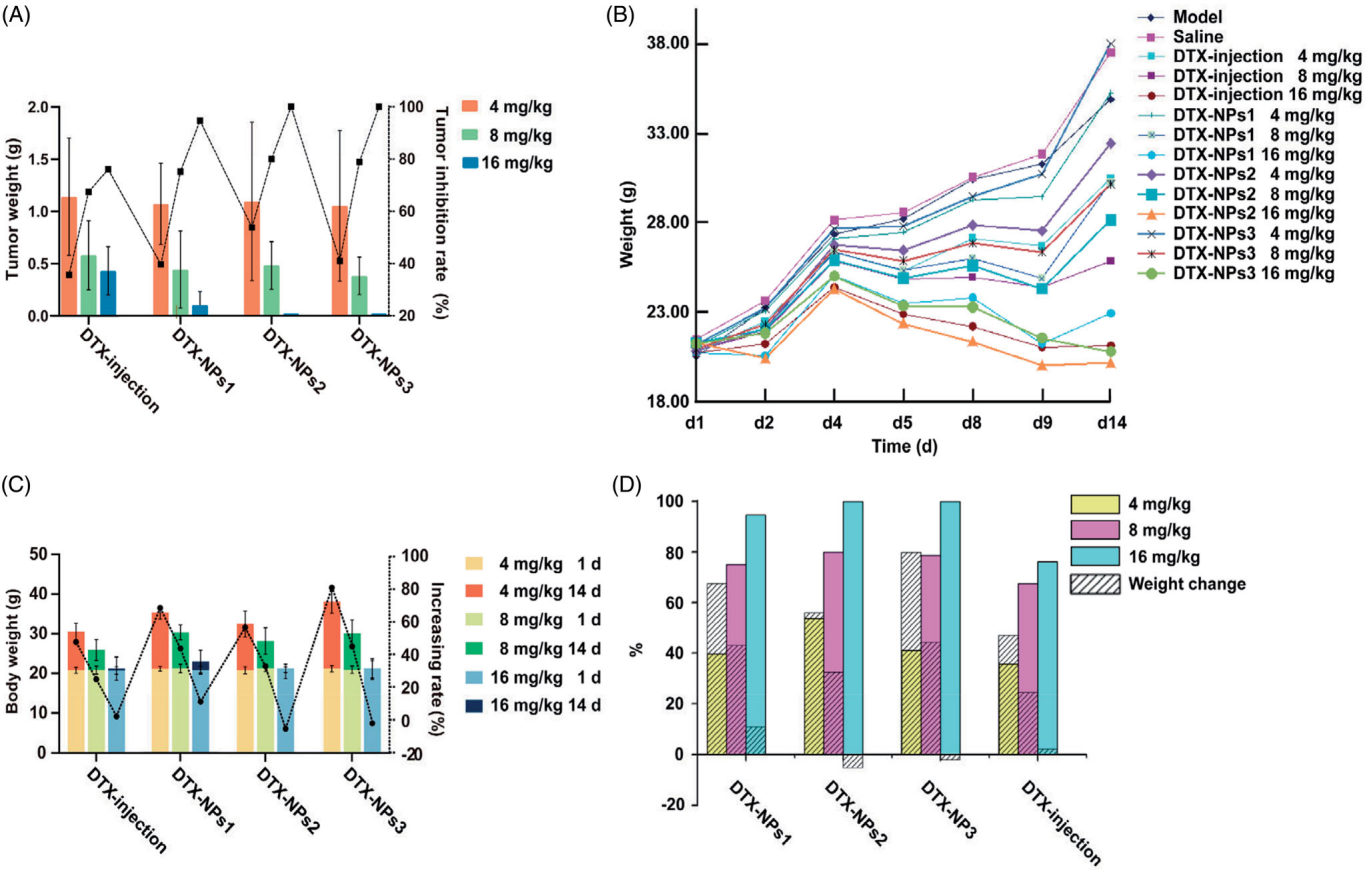
(A) The tumor weight and tumor inhibition rate of various formulations; (B) the changes of body weight of mice after intravenous injection of various formulations; (C) the body weight and its increasing rate of mice at the end of the experiment; (D) the contrast of body weight changes and tumor inhibition rate (*n* = 10).

Finally, the anti-tumor effect of DTX-NPs was evaluated on tumor-bearing mice. During the experiment, no obvious abnormality was found in the appearance, feeding, and activity state of the mice. The tumor weight and tumor inhibition rate of each experimental group were shown in Figure 5(A). The mean tumor weight of the model group was 1.78 ± 0.83 g, indicating that the tumor growth was normal. The anti-tumor effect of three DTX-NPs preparations was stronger than that of the commercial DTX-injection at the same dose. The tumor inhibition rates of different nanoparticles at low, medium and high doses were various. The experimental results showed that the tumor inhibition rates of three nanoparticles increased with dose, which indicated that they had a good dose-effect relationship. In addition, among three nanoparticle preparations, the tumor inhibition rate of DTX-NPs2 was significantly higher at the low dose than the other two preparations. While there was no obvious difference between the medium dose and high dose.

In terms of body weight change (Figure 5(B,C)), the weight growth rate of all administration groups was lower than the model group and the normal saline group, which illustrated that all preparations had varying degrees of toxic and side effects. It also could be found that the body weight gain decreased as the dosage increased, indicating the toxicity was dose-dependent. The final weight growth rate of three doses of DTX-NPs1 was higher than that of the DTX injection, indicating that the DTX NPs1 could reduce the side effects of DTX. A similar tendency also could be observed for low and medium doses groups of DTX-NPs2 and DTX-NPs3. In addition, comparing three DTX-NPs, the inhibitory effect on weight gain: DTX-NPs2 > DTX-NPs3 > DTX-NPs1, indicating that the nanoparticles prepared with mPEG_5000_PLA_15000_ had the lowest side effects.

Comprehensive Analysis of the inhibitory effects of nanoparticles on the body weight and tumor growth rate (Figure 5(D)), the tumor inhibition rates of three nanoparticles were significantly higher than those of the same dose of commercial injection, and the anti-tumor effect of middle-dose nanoparticles was similar to the high-dose commercial injection, which indicated that nanoparticles significantly improved the anti-tumor effect of DTX. The weight loss of most nanoparticles was less than that of the same dose of commercial injection, which indicated the nanoparticles could reduce the side effects of DTX. This could be explained by the change of drug distribution *in vivo* or the avoidance of Tween-80. These results demonstrated that due to the special physiological structure of the tumor vessels, nanoparticles could passively accumulate in the location of tumor, change the distribution of drugs in the body, and reduce the clearance rate of drugs in the blood to improve the bioavailability of drugs and increase treatment efficacy.

## 4. Conclusion

In this study, we prepared DTX-loaded nanoparticles by emulsion-solvent diffusion method, with mPEG-PLA as carrier materials. The formulation and preparation parameters were optimized by single-factor experiments. The final preparation method had good reproducibility, and the prepared DTX NP possessed a high encapsulation rate, high stability, and uniform particle size. The DTX NP could significantly improve the bioavailability of the drug both in healthy rats and tumor-bearing mice compared with commercially available injection. Moreover, it was found that the distribution of DTX in tumor tissues of the DTX-NPs was significantly higher than that of the DTX injection, indicating that the nano-carrier could increase the circulation time of drugs *in vivo* and promote the passive accumulation in tumor sites. Benefit from the superior pharmacokinetic profiles, the anti-tumor effect of the DTX-NPs was significantly better than the DTX injection. When the DTX-NPs administration dose was 16 mg/kg, the tumor inhibition rate almost reached 100%. In addition, during the administration period, the bodyweight increase of DTX-NPs groups was also higher than the DTX injection. Therefore, the DTX-loaded polymer-based nanoparticle prepared in this study holds great promise for sarcoma therapy application and clinical translation.
